# miR-582 Suppresses the Proliferation of B-Cell Precursor Acute Lymphoblastic Leukemia (BCP-ALL) Cells and Protects Them From Natural Killer Cell-Mediated Cytotoxicity

**DOI:** 10.3389/fimmu.2022.853094

**Published:** 2022-04-20

**Authors:** Xinxin Li, Yufei Zhang, Fei He, Dan Gao, Bo Che, Xiuli Cao, Siyong Huang, Minhua Zheng, Hua Han

**Affiliations:** ^1^ Xi’an Key Laboratory of Stem Cell and Regenerative Medicine, Institute of Medical Research, Northwestern Polytechnical University, Xi’an, China; ^2^ Research and Development Institute of Northwestern Polytechnical University in Shenzhen, Shenzhen, China; ^3^ State Key Laboratory of Cancer Biology, Department of Biochemistry and Molecular Biology, Fourth Military Medical University, Xi’an, China; ^4^ Department of Hepatobiliary Surgery, Xijing Hospital, Fourth Military Medical University, Xi’an, China; ^5^ Department of Medical Genetics and Developmental Biology, Fourth Military Medical University, Xi’an, China; ^6^ Department of Hematology, Xi’an International Medical Center Hospital, Xi’an, China

**Keywords:** BCP-ALL, miR-582, PPTC7, mitochondria, metabolism, NK cells, immune checkpoint, CD276

## Abstract

B-cell precursor acute lymphoblastic leukemia (BCP-ALL) is a malignancy characterized by the aberrant accumulation of immature B-cell precursors in bone marrow and other lymphoid organs. Although several intrinsic regulatory signals participating in BCP-ALL have been clarified, detailed intrinsic and extrinsic mechanisms that regulate BCP-ALL progression have not been fully understood. In the current study, we report that miR-582 is downregulated in BCP-ALL cells compared with normal B cells. Forced overexpression of miR-582 attenuated BCP-ALL cell proliferation and survival. We found that miR-582 overexpression disturbed the mitochondrial metabolism of BCP-ALL cells, leading to less ATP but more ROS production. Mechanistically, we identified PPTC7 as a direct target of miR-582. MiR-582 overexpression inhibited the activity of CoQ10, which is downstream of PPTC7 and played an important positive regulatory role in mitochondrial electron transportation. Finally, we found that overexpression of miR-582 upregulated the expression of immune checkpoint molecule CD276 and reduced NK cell-mediated cytotoxicity against BCP-ALL cells. CD276 blockade significantly increased NK cell-mediated cytotoxicity against miR-582-overexpressing BCP-ALL cells. Together, our research demonstrates that miR-582 acts as a negative regulator of BCP-ALL cells by reducing proliferation and survival, but protects BCP-ALL cells from NK cell-mediated cytotoxicity, suggesting that miR-582 may be a new therapeutic biomarker for BCP-ALL with CD276 blocker.

## Introduction

B-cell precursor acute lymphoblastic leukemia (BCP-ALL) is a type of hematopoietic malignancy found both in children and adults, and is characterized by the aberrant expansion of immature clonal B-cell precursors in bone marrow (BM), peripheral blood and lymphoid organs (1, 2). Although the prognosis of BCP-ALL has improved, the therapeutic effect of BCP-ALL patients with relapsed and drug-resistant remain unsatisfactory, and specifically, the 5-year survival rate for these types of patients is 35-40% (3). Previous studies have reported that during the development of B cells in BM, the disruption of the balance between pre-B cell proliferation and apoptosis is one of the important factors leading to the pathogenesis of BCP-ALL (4). Therefore, elucidating the regulatory mechanisms of BCP-ALL cells’ proliferation and survival is an important research focus for developing novel therapeutic approaches for BCP-ALL.

The progression of BCP-ALL is influenced by cell-intrinsic programs and the tumor microenvironment (TME) (5). In the TME of progressive BCP-ALL, anti-tumor immune cells, such as natural killer (NK) cells and CD8^+^ T cells, are prone to exhaust, lose or attenuate their cytotoxic activity against tumor cells, thus promoting tumor cell survival and proliferation (6, 7). Cell-intrinsic programs such as energy metabolism also play critical roles in regulating the survival and proliferation of BCP-ALL cells (8). For instance, electron transport chain (ETC) in mitochondria plays an essential role in energy metabolism, which is critically involved in the expansion of BCP-ALL cells (9). Specifically, the main function of ETC is transports electrons to generate ATP, which ultimately provides energy for cell survival and proliferation (10). Coenzyme Q10 (CoQ10), which is a crucial electron transporter of ETC, is involved in regulating the survival of B cells and T cells (11). Previous studies have shown that, compared with the control group, increasing the concentration of CoQ10 can promote the electron transport and improve the ATP synthesis, and reduce the generation of reactive oxygen species (ROS) (12). Protein phosphatase targeting CoQ7 (PPTC7), an important member of the protein phosphatase 2C (PP2C) family, has been reported to regulate the phosphorylation and dephosphorylation of protein complex-associated molecules which involved in electron transport (13) and CoQ10 production (14). Previous studied have shown that PPTC7 is a regulator of CoQ10 biosynthesis, and PPTC7/CoQ10 signaling facilitates ATP synthesis while preventing the accumulation of ROS, further promoting cell survival (14). However, whether PPTC7/CoQ10 signaling regulates BCP-ALL cell proliferation and survival, and the underlying regulatory mechanisms, remain unknown. Therefore, more studies are required to elucidate the exact regulatory mechanisms of PPTC7/CoQ10 signaling in BCP-ALL cell proliferation and survival.

miRNAs are endogenous ∼22 nt non-coding RNAs that downregulate protein expression by inhibiting target mRNA translation or promoting mRNA degradation (15). Previous reports indicated that miR-582 serves as an anti-oncogenic biomarker in many cancers, such as in intermediate risk AML (16), colorectal carcinoma (17). Recently, our lab found that miR-582 was highly expressed in murine pre-B cells, and knockout of miR-582 promotes the proliferation of murine pre-B cells (18). Relevant clinical studies also found that, compared with normal B cells, the expression of miR-582 is significantly lower in B cells of multiple sclerosis and B cells of dysregulated chronic lymphocytic leukemia (19, 20). MiR-582 expression in B cells from MLL rearranged pediatric acute lymphoblastic leukemia patients was significantly lower than that in other ALL patients (21). The above-mentioned research results indicated that dysregulated miR-582 expression may be involved in BCP-ALL progression. However, the specific regulation and mechanism of miR-582 in BCP-ALL needs further verification.

In the TME, miRNAs can not only regulate intracellular signaling pathways involved in cell survival and proliferation, but also alter the expression of immune checkpoint (IC) proteins on cell surface, such as PD-1/PD-L1 (22), CTLA-4 (23), CD276 (24), to subvert host immune surveillance. Recently, increasing evidence has indicated that IC molecules regulate the cytotoxic activity of T cells and NK cells. NK cells are important innate immune cells and have the ability to directly kill tumor cells without prior sensitization (25). Previous researches have shown that, in the TME, low expression of miR-29c and miR-142-5p upregulates the expression of some IC molecules such as CD276 and PD-L1, which further suppress the cytotoxic activity of NK cells and CD8^+^ T cells (26, 27). In diffuse large B cell lymphoma (DLBCL), overexpression of miR-5590-3p upregulates PD-L1 expression, and the high expression of PD-L1 on DLBCL cells promotes immune escape by inhibiting the cytotoxic activity of CD8^+^ T cells (28). However, whether miR-582 can regulate the expression of IC molecules on BCP-ALL cells and affect the cytotoxic activity of NK cells remains unknown. In this study, we show that overexpression of miR-582 inhibits the proliferation and survival of BCP-ALL cells. Moreover, we demonstrate that miR-582 directly regulates PPTC7/CoQ10 signaling to regulate BCP-ALL. We also showed that miR-582 overexpression promotes the expression of CD276 and protect BCP-ALL cells from NK cell-mediated cytotoxicity, which was reversed by anti-CD276 antibody.

## Materials and Methods

### Human Samples

BM samples were collected from BCP-ALL patients (n = 5) and non-BCP-ALL controls (n = 4, patients with unexplained anemia but excluded hematopoietic malignancies) hospitalized in the Department of Hematology, Xi’an International Medical Center Hospital, with signed informed consent and approved by the Ethics Committee of Fourth Military Medical University for use of human samples (**Supplementary Table S2**). B cells were enriched from BM by using MACSxpress B cell isolation kit (Miltenyi Biotec, USA) and erythrocyte depletion kit (Miltenyi Biotec, USA). The purity of the enriched B cells was > 90% as determined by using flow cytometry after CD19 staining. In some cases, B cells were further purified to > 99% purity by using FACS Aria II cell sorter (BD Biosciences, USA) after gating on CD19^+^ cells.

### Mice

NCG (NOD/ShiLtJGpt-*Prkdc^em26Cd52^Il2rg^em26Cd22^/*Gpt) mice (8 weeks old, female) were purchased from Gem Pharmatech (Nanjing, China). Mice were maintained in a specific pathogen-free (SPF) facility. All animal experiments were performed in accordance with the protocols approved by the Animal Experiment Administration Committee of the Fourth Military Medical University.

### Cell Culture, Infection, and Treatment

BCP-ALL cell lines (NALM-6, KOPN-8, and SUP-B15) and NK cells were obtained from the Beijing Beina Chuanglian Biotechnology Institute (Beijing, China). Specifically, NALM-6 and SUP-B15 cells were maintained in Roswell Park Memorial Institute 1640 (RPMI-1640) medium (Invitrogen, USA) supplemented with 10% FBS, 2 mM L-glutamine, 100 U/mL penicillin and 100 μg/mL streptomycin. KOPN-8 cells were maintained in Iscove’s Modified Dulbecco Medium (IMDM) (Invitrogen, USA) supplemented with 10% FBS, 2 mM L-glutamine, 100 U/mL penicillin and 100 μg/mL streptomycin and incubated at 37°C in 5% CO_2_ and 95% air. NK cells were maintained in complete medium with 1000 U/ml recombinant human IL-2. Cells were routinely tested for the absence of mycoplasma using MycoAlertTM PLUS Mycoplasma Detection Kit (Lonza, USA).

In order to test the infection efficiency of lentivirus on BCP-ALL cells, we infect cells with EGFP-labeled lentivirus (MOI of 60), after 24 hours (h), we tested the infection efficiency by flow cytometry, and found that the infection efficiency of BCP-cells > 99% (**Supplementary Figure S1A**). Then, to overexpress human single-stranded mature miR-582 in BCP-ALL cells, a synthetic precursor miR-582 [pre-miR-582] gene fragment was inserted into the lentiviral vector GV309. After the lentivirus package and infection of BCP-ALL cells, pre-miR-582 was expressed and further cleaved by Dicer to form single-stranded, mature miR-582. Specifically, for infection, cells (1 × 10^6^/well) were seeded in 96-well plates. Lentivirus particles were added at a multiplicity of infection (MOI of 60), and cultured for 12 h, then the medium was changed and cultured for 72h according to experimental designs. In some experiments, PPTC7 overexpressing lentivirus (MOI of 60), PPTC7 overexpressing lentivirus (MOI of 60) with pre-miR-582 overexpressing lentivirus (MOI of 60) was used to infect BCP-ALL cells. In some experiments, the anti-CD276 antibody (100ng/well, MGA271) was added to the cultures.

### Cell Proliferation and Apoptosis

Cell proliferation and apoptosis assays were performed as described previously (18). Specifically, after infection with lentivirus for 48 h, NALM-6, KOPN-8 and SUP-B15 cells (1 × 10^6^/well) were resuspended in PBS in tubes and incubated with 5,6-carboxyfluorescein diacetate succinimidyl ester (CFSE) (Biolegand, USA) at the concentration of 3 μM for 20 min in cell incubator, followed by adding 10% FBS-containing medium to quench CFSE. After washing twice by using10% FBS medium, cells were resuspended in culture medium and cultured in 96-well U bottom plates for another 24 h, and the proliferation was analyzed by flow cytometry.

To evaluate apoptosis, cells (1 × 10^6^ cells/well) were resuspended in 100 μl binding buffer, prior to the addition of 5 μL Annexin V (Biolegand, USA) and incubated for 15 min. Then, cells were washed twice with the binding buffer, and 200 μL of binding buffer and 5 μL 7-AAD were added. The percentage of live cells, early and lately apoptotic cells were analyzed by flow cytometry.

### ATP Measurement

ATP level was determined by using an ATP assay kit (Beyotime Biotechnology, China) according to the manufacturer’s instructions. Briefly, cells were broken by the lysis buffer, and the supernatant was gathered and mixed with the ATP detecting solution. The ATP level was detected by firefly luciferase activities with a luciferase assay system (Promega, USA).

### Extracellular Flux Analysis

Extracellular flux analyses were carried out with a Seahorse XF-24 analyzer. BCP-ALL cells (5 × 10^5^/well) are adhere-cultured with PLL-coated plate for 12 h. Then, cells were washed three times in the Seahorse assay medium. Mitochondrial metabolism (OCRs) was analyzed with Seahorse Mito Test kits (Agilent, Waldbronn, Germany) according to the manufacturer’s instructions.

### ROS Generation Assay

For cellular total ROS detect, 2’,7’-dichlorofluorescin diacetate (DCFH-DA, Beyotime, China) was diluted by serum-free medium to a concentration of 10 μM. Then, cells were re-suspended in DCFH-DA medium and incubated at 37°C for 30 min. For mitochondrial ROS detect, cells were re-suspended in medium containing 10 μM DCFH-DA and 25 nM MitoTacker (Invitrogen, USA), and incubated at 37°C for 30 min. Cells were washed twice with serum-free medium and analyzed by flow cytometry.

### Glucose Uptake Assay

For glucose uptake assay, cells were pelleted at 500 g for 5 min at 4°C and then washed twice with glucose-free medium. The 2-deoxy-2-[(7-nitro-2,1,3-benzoxadiazol-4-yl)amino]-D-glucose (2-NBDG, Cayman Technologies, USA) was diluted to the concentration of 150 μg/mL in glucose-free medium. Then, the 2-NBDG dilution (100 μL) was added to the glucose-free medium and incubated at 37°C for 30 min. Finally, the cells were washed twice with PBS to remove the residual 2-NBDG and analyzed by flow cytometry.

### RNA-Seq Analysis

NALM-6 cells were infected with pre-miR-582 lentivirus or pre-miR-Ctrl lentivirus for 72 h, and total cellular RNA was isolated using the TRIzol reagent. RNA was reverse-transcribed into cDNA for constructing the library. Then, RNA sequencing was conducted with the cDNA library. RNA sequencing using Illumina Novaseq6000 by Gene Denovo Biotechnology Co. (Guangzhou, China). The raw reads were filtered, and clean reads were mapped to Ensembl_release103 whole genome using HISAT2.2.4. The mapped reads of each sample were assembled by using StringTie v1.3.1 in a reference-based approach. For each transcription region, a FPKM (fragment per kilobase of transcript per million mapped reads) value was calculated to quantify its expression abundance and variations, using the StringTie software. Original RNA-seq data have been deposited in the NCBI (https://www.ncbi.nlm.nih.gov/sra/PRJNA811525). The online tools (http://www.omicshare.com/tools) were used for the subsequent bioinformatic analysis.

### Human BCP-ALL Xenograft Mouse Model

NCG mice were inoculated with 1 × 10^6^ NALM-6 cells which infected with lentivirus expressing pre-miR-Ctrl or pre-miR-582 together with luciferase through tail veins (day 0). On day 14 and 21, tumor burden was determined by IVIS^®^ Spectrum *In Vivo* Imaging System in each mouse. BM and spleen cells were collected on day 21, and the proportion and number of human CD19^+^ NALM-6 cells were detected by flow cytometry. Finally, the survival days of tumor-bearing mice were recorded and a survival curve was drawn.

### Cytotoxicity Assay

Cytotoxicity assays were performed as described previously (25). NALM-6, KOPN-8 and SUP-B15 cells were co-cultured with the effector NK cells in a 96-well V-bottom plate at 37°C for 4 h. At the end of the co-culture, cells were collected and incubated with anti-human CD56, anti-human CD107a antibodies against surface markers to label NK cells and the degranulation marker of NK cells. Cells were then washed and resuspended in Cytofix/Cytoperm solution (BD Biosciences) at 4°C for 30 min. Fixed and permeabilized cells were stained with anti-human Granzyme B (GZMB) antibody for analysis by flow cytometry. Data were analyzed using FlowJo V10 software (Tree Star, Ashland, OR).

### Statistical Analysis

GraphPad Prism 7.0 software (GraphPad Software, USA) and SAS8e software were used for statistical analyses. Student’s t-test or paired t-test was used to compare two independent or paired groups. Kaplan-Meier method was used to estimate survival functions and log-rank test was used to compare any two survival curves. All results are expressed as mean ± SD. *P <* 0.05 was considered statistically significant.

## Results

### miR-582 Is Downregulated in BCP-ALL Cells

**Figure 1:**
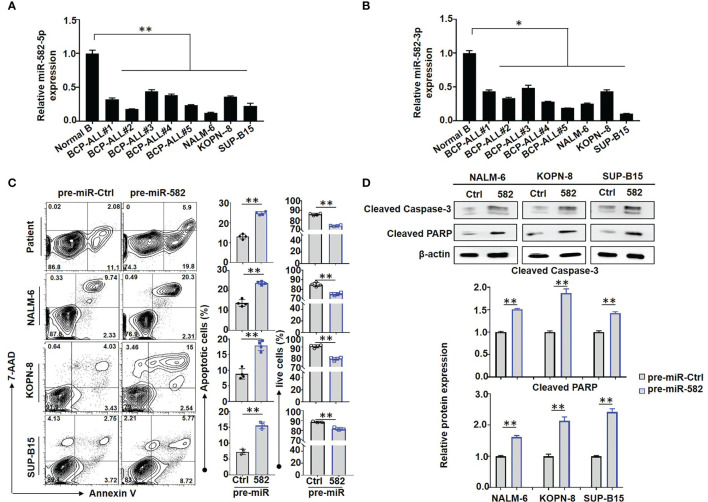
Overexpression of miR-582 inhibits the survival of BCP-ALL cells. **(A, B)** Relative expression of human miR-582-5p and miR-582-3p in normal B cells, BCP-ALL patients B cells, NALM-6, KOPN-8 and SUP-B15 cells were determined by qRT-PCR. **(C)** BCP-ALL patients B cells, NALM-6, KOPN-8 and SUP-B15 cells were infected with pre-miR-582 lentivirus or their control lentivirus, and cultured for 72 h. Apoptosis was evaluated by using flow cytometry after staining of Annexin V and 7-AAD. The percentages of infected live (Annexin V^-/^7-AAD^-^) BCP-ALL cells, infected early and late apoptotic (Annexin V^+^/7-AAD^-/+^) BCP-ALL cells were quantitatively compared (n = 3-4). **(D)** BCP-ALL cells were infected with pre-miR-582 lentivirus or their control lentivirus for 72 h, and Cleaved Caspase-3, Cleaved PARP proteins were determined by Western blotting, with β-actin as an internal control (n = 3). Bars represent means ± SD, *P < 0.05, **P < 0.01.

Aberrant expression of miR-582 has been implicated in several solid tumors including non-small cell lung cancer (29, 30), prostate cancer (31), and hepatocellular carcinoma (32). Our lab recently found that miR-582 represses pre-B proliferation in early B cell development in mice (18). To evaluate whether miR-582 regulates BCP-ALL progression, we examined the expressions of miR-582-5p and miR-582-3p in B cells from BCP-ALL patients or controls, and BCP-ALL cell lines NALM-6, KOPN-8, and SUP-B15. The results showed that, compared with the controls, miR-582-5p and miR-582-3p were downregulated in B cells from BCP-ALL patients and the BCP-ALL cell lines (**Figures 1A, B** **)**, suggesting that miR-582 is potentially involved in BCP-ALL.

### miR-582 Inhibits the Proliferation and Promotes the Apoptosis of BCP-ALL Cells

**Figure 2:**
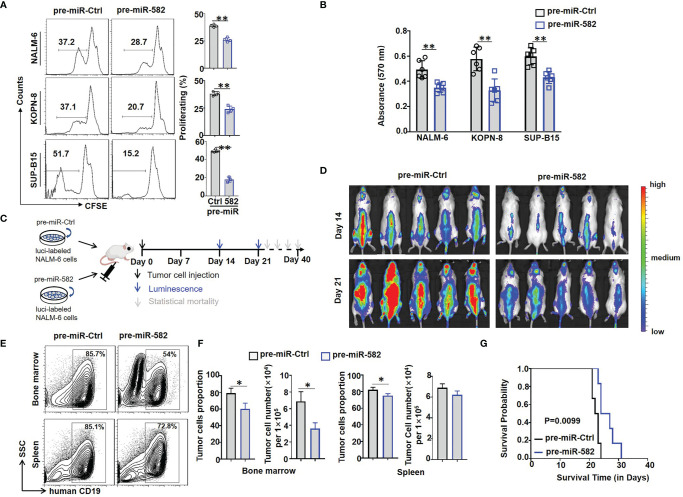
Overexpression of miR-582 inhibits the proliferation of BCP-ALL cells. **(A)** NALM-6, KOPN-8 and SUP-B15 cells were infected with pre-miR-582 lentivirus or their control lentivirus. Cell proliferation of infected cells was determined using the CFSE labeling assay (n = 3-6). **(B)** NALM-6, KOPN-8 and SUP-B15 cells were infected with pre-miR-582 lentivirus or their control lentivirus, and cultured for 72 h. Cell viability was detected by MTT method (n = 6). **(C, D)** Experimental timeline for *in vivo* study. luciferase (luci)-labeled NALM-6 were infected with pre-miR-582 lentivirus or their control lentivirus, then, an orthotopic xenograft BCP-ALL mouse model was established by i.v injecting 1 × 10^6^ luci-labeling pre-miR-582 NALM-6 cells or pre-miR-Ctrl NALM-6 cells into NCG mice on day 0. Tumor burden was determined on day 14 and 21. **(E, F)** On Day 21, single cell suspensions of BM and spleen from BCP-ALL mouse model were analyzed by flow cytometry **(E)**, and the percentage of human CD19^+^ B cells were quantitatively compared **(F)** (n = 3). **(G)** Survival of NCG mice which injected with miR-582-overexpressing NALM-6 cells and control NALM-6 cells. Bars represent means ± SD, *P < 0.05, **P < 0.01.

We next accessed the function of miR-582 in BCP-ALL cells. BCP-ALL cells were infected with pre-miR-582 or pre-miR-Ctrl (control) lentivirus, firstly, we evaluate the overexpression efficiency by using qRT-PCR method, the results showed that, compared with control group, the expression of miR-582-5p is significantly increased in pre-miR-582 group (**Supplementary Figure S1B**). Then we stained with Annexin V and 7-AAD prior to flow cytometry. The result showed that, compared with pre-miR-Ctrl infected cells, the proportion of apoptosis (Annexin V^+^/7-AAD^+/-^) cells was significantly increased and the proportion of live (Annexin V^-^/7-AAD^-^) cells was significantly decreased in pre-miR-582 infected cells **(** **Figure 1C** **)**. Western blotting confirmed that overexpression of miR-582 significantly increased the expression of Cleaved caspase-3 and Cleaved PARP proteins, two makers of apoptosis (33, 34) **(** **Figure 1D** **)**. We also examined the role of miR-582 in regulating BCP-ALL proliferation. BCP-ALL cells were infected with pre-miR-582 or control lentivirus for 48 h, and labeled with CFSE for another 24 h prior to flow cytometry analysis. The result showed that, compared with the control infected cells, the proportion of CFSE^low/-^ BCP-ALL cells in pre-miR-582 infected cells was significantly decreased **(** **Figure 2A** **)**. By using the MTT assay, we found that, compared with control group, the cell viability and proliferation ability in pre-miR-582 infected BCP-ALL cells were significantly reduced, which confirming the above data **(** **Figure 2B** **)**. These results suggested that miR-582 inhibits the proliferation and promotes the apoptosis of BCP-ALL cells.

Next, firefly luciferase-expressing NALM-6 cells were infected with pre-miR-582 or control lentivirus for 24 h, and cells were injected intravenously (i.v) into NCG mice to establish an orthotopic xenograft NALM-6 model. Tumor growth was monitored by bioluminescence imaging, starting at day 14 after the infusion of tumor cells (**Figure 2C**). The results showed that, compared with the control group, tumor progression in the pre-miR-582 infected group was significantly inhibited on day 14 and 21 **(** **Figure 2D** **)**. Consistently, the proportion and number of tumor cells in BM and spleen were significantly decreased in pre-miR-582 infected group as compared with the control **(** **Figures 2E, F** **)**. The survival of tumor-bearing mice was significantly longer in the pre-miR-582 infected group than the control group **(** **Figure 2G**).

### miR-582 Inhibits Mitochondrial Energy Metabolism of BCP-ALL Cells

**Figure 3:**
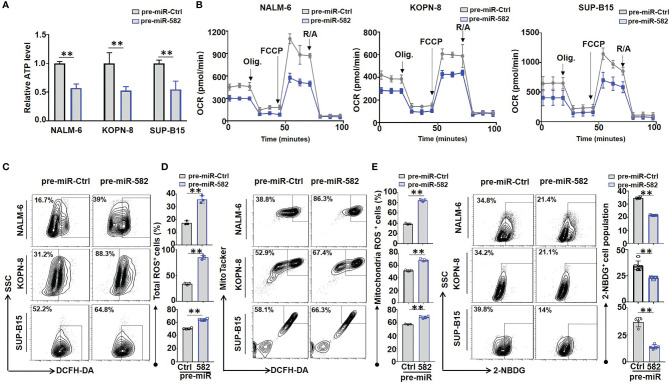
Overexpression of miR-582 inhibits mitochondrial energy metabolism of BCP-ALL cells. **(A)** After infection with pre-miR-582 lentivirus or their control lentivirus for 72 h, the contents of ATP in NALM-6, KOPN-8 and SUP-B15 cells were measured by using a luciferase reporter assay system. The luminescence level was normalized to pre-miR-Ctrl group in each individual (n = 3). **(B)** NALM-6, KOPN-8 and SUP-B15 cells were infected with pre-miR-582 lentivirus or their control lentivirus for 72 h, the mitochondrial metabolic pressure was detected by using XF Extracellular Flux Analyzer (n = 3). **(C, D)** NALM-6, KOPN-8 and SUP-B15 cells were infected with pre-miR-582 lentivirus and their control lentivirus for 72 h, the total intracellular ROS and mitochondria ROS were determined by flow cytometry, total intracellular ROS was labeled by DCFH-DA, the mitochondria ROS was labeled by MitoTacker and DCFH-DA (n = 3-5). **(E)** NALM-6, KOPN-8 and SUP-B15 cells were infected with pre-miR-582 lentivirus or their control lentivirus for 72 h, 2-NBDG was added to glucose-free medium for 30 min and intracellular 2-NBDG was analyzed by flow cytometry and quantitatively by statistics the proportion of 2-NBDG^+^ cells (n = 3-5). Bars represent means ± SD, **P < 0.01.

Previous studies have demonstrated that mitochondrial energy metabolism plays an important role in regulating BCP-ALL cell survival and proliferation (8). We next tested whether mitochondrial energy metabolism is involved in regulating BCP-ALL cell proliferation and survival by miR-582. We found that overexpression of miR-582 significantly reduced the content of ATP in BCP-ALL cells **(** **Figure 3A** **)**. We then used the extracellular flux analyses with a Seahorse device to detect the O_2_ consumption rate (OCR) of BCP-ALL cells. The result showed that overexpression of miR-582 reduced the OCR (the basal respiration, ATP production and maximal respiration) of BCP-ALL cells **(** **Figure 3B** **)**. However, the spare respiratory capacity did not decrease significantly in pre-miR-582-infected BCP-ALL cells, except in NALM-6 cells **(** **Figure 3B** and **Supplementary Figure S3** **)**. These results suggested that miR-582 inhibits mitochondrial energy metabolism of BCP-ALL cells, but there is no difference in the ability of BCP-ALL cells to respond to the demand for spare respiratory capacity, except in NALM-6 cells.

Next, we investigated the effect of miR-582 on ROS production in BCP-ALL cells, the results showed that miR-582 overexpression significantly increased total and mitochondrial ROS production in BCP-ALL cells as compared with the control **(** **Figures 3C, D** **;** **Supplementary Figure S2** **)**. Moreover, compared with the control, overexpression of miR-582 significantly inhibit the glucose uptake in BCP-ALL cells **(** **Figure 3E** **)**. These results indicated that miR-582 overexpression inhibits mitochondrial energy metabolism and promotes ROS production in BCP-ALL cells.

### miR-582 Downregulates PPTC7 in BCP-ALL Cells

**Figure 4:**
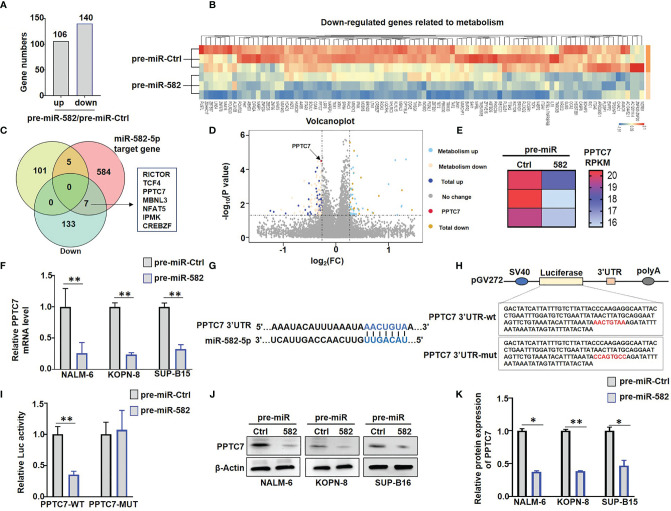
miR-582 downregulates PPTC7 in BCP-ALL cells. **(A)** Significantly different expressed genes (differentially upregulated genes and differentially downregulated genes) in pre-miR-582 NALM-6 cells/pre-miR-Ctrl NALM-6 cells**. (B)** 94 genes which related to metabolism are significantly downregulated in pre-miR-582 NALM-6 cells. **(C)** 7 significantly downregulated metabolism-related genes were predicted target genes of human miR-582-5p by combined with RNA-seq data and TargetScan 6.2 database. **(D, E)** The mRNA expression of PPTC7 in pre-miR-Ctrl NALM-6 cells and pre-miR-582 NALM-6 cells were showed by volcano plot and heatmap. **(F)** NALM-6, KOPN-8 and SUP-B15 cells were infected with pre-miR-582 lentivirus or their control lentivirus for 72 h, and PPTC7 mRNA were determined by qRT-PCR, with β-actin as an internal control (n = 3). **(G)** Alignment of the seed sequence of human miR-582-5p with human PPTC7 3’UTR. Complementary bases are marked with blue color. **(H)** The sequences of PPTC7 3’UTR-wt and PPTC7 3’UTR-mut used for schematic of the reporter constructs. **(I)** HEK293T cells were transfected with miR-582-5p and different 3’UTR reporters of human PPTC7 for 72 h. Luciferase activity in cell lysates were determined by the dual luciferase reporter assay (n = 3). **(J, K)** NALM-6, KOPN-8 and SUP-B15 cells were infected with pre-miR-582 lentivirus or their controls for 72 h, and PPTC7 protein were determined by Western blotting, with β-actin as an internal control (n = 3). Bars represent means ± SD, *P < 0.05,**P < 0.01.

To address the molecular mechanism of miR-582 regulating energy metabolism in BCP-ALL cells, we compared the transcriptomes of NALM-6 cells infected with pre-miR-582 or control lentivirus. The result showed that miR-582 overexpression upregulated the mRNA expression of 106 genes and downregulated 140 genes **(** **Figure 4A** **).** Among the 140 downregulated genes, 94 genes are metabolism-related genes **(** **Figure 4B** **)**. Further analyses of RNA-seq data and potential miR-582 targets using the TargetScan 6.2 database identified 7 metabolism-related and downregulated genes as predicted target genes of human miR-582-5p **(** **Figure 4C** **)**, including PPTC7, a gene reported to play an important role in mitochondrial energy metabolism **(** **Figures 4D, E** **)**. QRT-PCR confirmed that infection of the pre-miR-582 lentivirus significantly downregulated the mRNA expression of PPTC7 in all three BCP-ALL cell lines **(** **Figure 4F** **)**.

The 3’UTR of PPTC7 contains a miR-582-5p recognition site. We cloned the PPTC7 3’UTR fragment harboring the miR-582-5p binding site, and constructed reporter genes with the wild type of fragment or the fragment with the mutated binding site **(** **Figures 4G, H** **)**. Transfection of miR-582-5p mimics into HEK-293T cells significantly suppressed the wild-type (WT) reporter activity but failed to suppress the reporter with the mutated (MUT) PPTC7 3’UTR **(** **Figure 4I** **)**. Moreover, we found that overexpression of miR-582 significantly inhibited the protein expression of PPTC7 in NALM-6, KOPN-8 and SUP-B15 cells **(** **Figures 4J, K** **)**. Taken together, these results verified that miR-582 directly targets the 3’UTR of PPTC7 to inhibit PPTC7 expression in BCP-ALL cells.

### miR-582 Attenuates Mitochondrial Energy Metabolism by Inhibiting PPTC7/CoQ10 Signaling in BCP-ALL Cells

**Figure 5:**
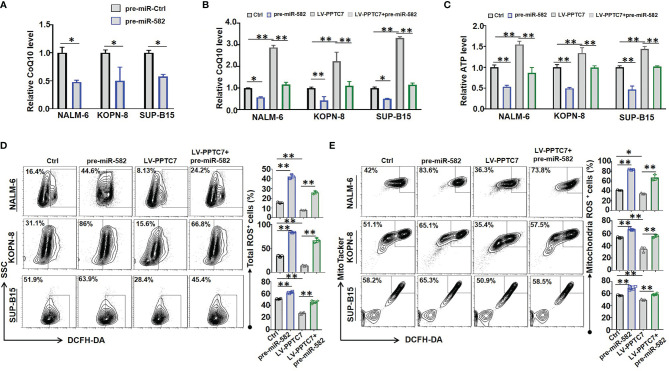
miR-582 attenuates BCP-ALL cells mitochondrial energy metabolism by inhibiting PPTC7/CoQ10 signaling. **(A)** NALM-6, KOPN-8 and SUP-B15 cells were infected with pre-miR-582 lentivirus or their control lentivirus for 72 h, CoQ10 contents in cell lysates was evaluated and compared (n = 3). **(B)** NALM-6, KOPN-8 and SUP-B15 cells were infected with PPTC7 overexpression lentivirus (LV-PPTC7), with/without cultured with pre-miR-582 lentivirus for 72 h. CoQ10 contents in cell lysates was evaluated and compared (n = 3). **(C)** NALM-6, KOPN-8 and SUP-B15 cells were infected with LV-PPTC7, with/without cultured with pre-miR-582 lentivirus for 72 h, relative ATP level in cell lysates was evaluated and compared. **(D, E)** NALM-6, KOPN-8 and SUP-B15 cells were infected with LV-PPTC7, with/without cultured with pre-miR-582 lentivirus for 72 h, the total ROS and Mitochondria ROS were determined by flow cytometry, total intracellular ROS was labeled by DCFH-DA, the mitochondria ROS was labeled by MitoTacker and DCFH-DA. Bars represent means ± SD, *P < 0.05, **P < 0.01.

To further evaluate the role of PPTC7 in miR-582-mediated mitochondrial energy metabolism, we examined the effects of miR-582 overexpression on CoQ10 production, which is reported to be a downstream molecule of PPTC7 in Hela cells (14). BCP-ALL cells were infected with pre-miR-582 or control lentivirus for 72 h, and CoQ10 content was examined. The results showed that miR-582 overexpression inhibited the CoQ10 production in the three BCP-ALL cell lines (**Figure 5A**). We then infected BCP-ALL cells with PPTC7 overexpression lentivirus, and found that PPTC7 overexpression strongly promotes the mitochondrial energy metabolism, including promote the production of CoQ10 and ATP, inhibit the production of ROS (**Figures 5B–E**), which demonstrated that PPTC7 positively regulate mitochondrial energy metabolism through COQ10 in BCP-ALL cells. However, compared with the PPTC7 overexpression group, simultaneous overexpression of PPTC7 and miR-582 partially suppressed the increase in COQ10 and ATP production, and promoted the generation of ROS (**Figures 5B–E**). These results suggested that miR-582 attenuates mitochondrial energy metabolism of BCP-ALL cells *via* inhibiting PPTC7/CoQ10 signaling.

### miR-582 Overexpression Protects BCP-ALL Cells From NK Cell-Mediated Cytotoxicity

**Figure 6:**
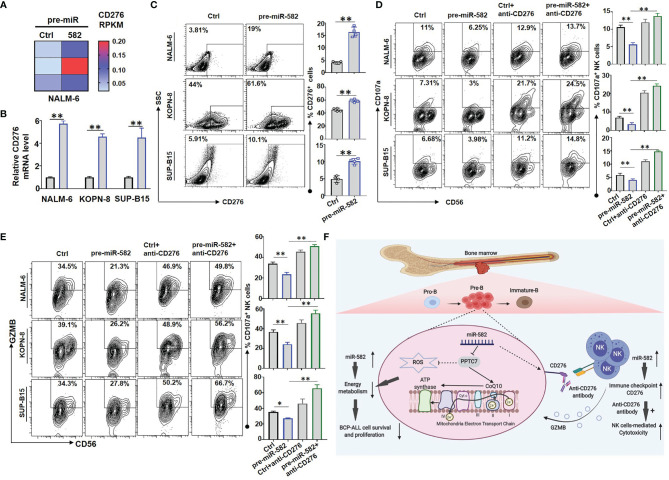
Overexpression of miR-582 protects BCP-ALL cells from NK cell-mediated cytotoxicity. **(A)** NALM-6 cells were infected with pre-miR-582 lentivirus or its control lentivirus for 72 h, and CD276 mRNA were determined by RNA-seq. **(B, C)** NALM-6, KOPN-8 and SUP-B15 cells were infected with pre-miR-582 lentivirus or its control lentivirus for 72 h, and CD276 mRNA were determined by qRT-PCR **(B)**; the proportion of CD276^+^ cells were determined by flow cytometry **(C)**. **(D, E)** NALM-6, KOPN-8 and SUP-B15 cells were infected with pre-miR-582 lentivirus and their control lentivirus, cultured with or without anti-CD276 antibody, and co-cultured with NK cells for 4 h, the proportion of CD107a^+^ NK and GZMB^+^ NK cells were determined by flow cytometry. **(F)** The signaling pathway of miR-582 regulate the progression of BCP-ALL and protect BCP-ALL cells from natural killer cell-mediated cytotoxicity, figure created with BioRender.com. Bars represent means ± SD, *P < 0.05, **P < 0.01.

The IC molecules, such as PD-1, CTLA-4 and CD276, inhibit the cytotoxic activity and promote the exhaustion of NK cells and T cells (26, 27). RNA-seq results of NALM-6 cells showed that, compared with the control, miR-582 overexpression significantly upregulated the mRNA expression of CD276, which is an important IC molecule inhibiting NK-mediated cytotoxicity (26) **(** **Figure 6A** **)**. Then, three BCP-ALL cell lines were infected with pre-miR-582 and control lentivirus for 72 h, and the expression of CD276 was examined. The results showed that, compared with the control, miR-582 overexpression significantly increased the mRNA and protein expression of CD276 **(** **Figures 6B, C** and **Supplementary Figure S4A** **)**. We further determined whether miR-582 overexpression could protect BCP-ALL cells from NK cell-mediated cytotoxicity by upregulating CD276. The cytotoxicity assay showed that, compared with the control group, the expression of CD107a and GZMB was significantly reduced in NK cells co-cultured with miR-582-overexpressing BCP-ALL (NALM-6, KOPN-8, as well as SUP-B15) cells **(** **Figures 6D, E** **;** **Supplementary Figures S4B, C** **)**. Treat with anti-CD276 antibody significantly increased the NK cell-mediated cytotoxicity on BCP-ALL cells with miR-582 overexpression **(** **Figures 6D, E** **)**. These results suggested that miR-582 upregulates CD276 to protect BCP-ALL cells from NK cell-mediated cytotoxicity.

## Discussion

In this study, we report that miR-582 regulates BCP-ALL progression by negatively regulating the survival and proliferation of BCP-ALL cells. We have identified that the PPTC7, which regulates CoQ10 production and mitochondrial energy metabolism, is the downstream target of miR-582 involved in regulating the survival and proliferation of BCP-ALL cells. We also found that miR-582 overexpression promotes the expression of CD276 and protects BCP-ALL cells from NK cell-mediated cytotoxicity, which can be overcome by CD276 blockade with a specific antibody **(** **Figure 6F** **)**. Our observations in clinical samples suggest that miR-582 is downregulated in BCP-ALL, therefore these findings provide a new molecular mechanism of BCP-ALL progression, and suggest that low miR-582 may provide progression advantages in human BCP-ALL, which may insensitive to anti-CD276 therapy.

Previous studies have established a link between miR-582 and tumor procession (30, 35). For instance, in hematopoietic malignancies and solid tumors, such as acute myeloid leukemia (36), chronic lymphocytic leukemia (37), multiple myeloma (38), bladder cancer (39), and human colorectal carcinoma (17), miR-582 serves as an anti-oncogenic biomarker and can inhibit proliferation and induce apoptosis of these malignant cells by targeting different genes, such as cyclin B2, HNRNPA1, HMGB2 and Rab27a (17, 35–38). These studies have shown that miR-582 plays a negative regulatory role in tumor progression. Recently, miR-582 has also been found to participate in regulating several energy metabolism-related genes, such as ERO1A (40) and AKT/mTOR signaling (41), in different biological processes. Our lab has found that miR-582 is highly expressed in murine pre-B cells, and knockout of miR-582 promotes pre-B cell proliferation, while overexpression of miR-582 inhibits pre-B cell proliferation (18). Consistently, in this study, we revealed an anti-oncogenic role of miR-582 in BCP-ALL. These findings indicated that miR-582 may serve as an important molecule in BCP-ALL progression, and miR-582 overexpression in BCP-ALL cells could be a potential strategy for inhibit BCP-ALL progression.

Tumor cells are characterized by extensive proliferation, in which mitochondrial energy metabolism plays an important role (42). Previous researches have shown that improving mitochondrial energy metabolism promotes the survival and proliferation of BCP-ALL cells (8). A recent study further found that mitochondrial energy metabolism was significantly decreased in PPTC7 downregulated cells, resulting in decreased ATP synthesis and increased ROS production (13), indicating that PPTC7 plays a key role in the mitochondrial energy metabolism pathway of cells. In our study, we have provided evidence that miR-582 downregulates mitochondria energy metabolism at least partially *via* directly targeting PPTC7, which contributes to attenuated the survival and proliferation of BCP-ALL cells. Moreover, a previous research has shown that CoQ10, which is an crucial downstream molecule of PPTC7, facilitates ATP synthesis while prevents the accumulation of ROS, further promoting cell survival (14). In our study, we have provided functional evidence that miR-582 negatively regulates CoQ10 synthesis through inhibiting PPTC7, resulting in less ATP synthesis and more ROS production. Therefore, we suggest that miR-582 downregulates PPTC7 and reduces CoQ10 production by directly targeting PPTC7, and thereby functions as a novel negative regulator of the electron transport chain in mitochondrial energy metabolism of BCP-ALL cells.

During the progression of various tumors, tumor cells in the TME often tend to promote the exhaustion of anti-tumor immune cells, such as NK cells, by upregulating the expression of IC molecules, to reduce cytotoxicity and enhance immune escape (43). Previous researches have shown that CD276, an important IC molecule, is often highly expressed in neuroblastoma (44) and non-small cell lung cancer cells (45), inhibits the cytotoxicity of NK cells. CD276-CAR NK cells or blocking CD276 with anti-CD276 antibody often rescues the anti-tumor ability of NK cells (44–46). A previous study showed that miR-29c negatively regulates CD276 expression in tumor cells (43). However, in our research, we found that miR-582 positively regulates the CD276 expression in BCP-ALL cells, resulting in reduced NK cell-mediated cytotoxicity to BCP-ALL cells. The specific mechanism of miR-582 in regulating the expression of CD276 is still unknown. miR-582-mediated metabolic remodeling could be involved in, but more studies are required to access this question. Our study further suggests that CD276 blockade can relieve the inhibition of NK cell-mediated cytotoxicity likely in BCP-ALL cells with high miR-582 expression, and therefore miR-582 may serve as a biomarker for anti-CD276 therapy of BCP-ALL.

In conclusion, our research reported here has uncovered a role of miR-582 as a negative regulator of human BCP-ALL cells proliferation and survival. Our findings provide novel insights into how miR-582 inhibits the proliferation and survival of BCP-ALL cells by targeting PPTC7 to reduce CoQ10 level and further inhibit mitochondrial energy metabolism in BCP-ALL cells. miR-582 also promotes the expression of CD276 and protect BCP-ALL cells from NK cell-mediated cytotoxicity, which might of significance in treatment of BCP-ALL with anti-CD276 antibodies.

## Data Availability Statement

The datasets presented in this study can be found in online repositories. The names of the repository/repositories and accession number(s) can be found below: NCBI - PRJNA811525.

## Ethics Statement

The studies involving human participants were reviewed and approved by the Ethics Committee of Fourth Military Medical University for use of human samples. Written informed consent to participate in this study was provided by the participants’ legal guardian/next of kin.

## Author Contributions

Conception and design: MZ and HH. Performing methodology: XL, YZ, FH, and BC. Analysis of data: DG and XC. Critical materials: XL and SH. Writing manuscript: XL and HH. Study supervision: HH. All authors contributed to the article and approved the submitted version.

## Funding

This work was supported by Natural Science Basic Research Program of Shaanxi (Program No. 2020JQ-147), Guangdong Basic and Applied Basic Research Foundation (2020A1515110094) and the Fundamental Research Funds for the Central Universities of China (3102019YX01004), and National Natural Science Foundation (31730041, 91339115, 31671523) of China.

## Conflict of Interest

The authors declare that the research was conducted in the absence of any commercial or financial relationships that could be construed as a potential conflict of interest.

## Publisher’s Note

All claims expressed in this article are solely those of the authors and do not necessarily represent those of their affiliated organizations, or those of the publisher, the editors and the reviewers. Any product that may be evaluated in this article, or claim that may be made by its manufacturer, is not guaranteed or endorsed by the publisher.
