# Supplementary material for: miR-582 Suppresses the Proliferation of B-Cell Precursor Acute Lymphoblastic Leukemia (BCP-ALL) Cells and Protects Them From Natural Killer Cell-Mediated Cytotoxicity
Source: Front Immunol. 2022 Apr 20;13:853094. doi: 10.3389/fimmu.2022.853094 (PMC9065596; doi:10.3389/fimmu.2022.853094)
Supplement: Supplementary file 1 [file DataSheet_1.docx]

**Supplementary Materials**

**Supplementary Materials and Methods**

**Quantitative RT-PCR analysis**

Quantitative RT-PCR (qRT-PCR) analysis was performed as described previously. Total RNA was isolated using the Trizol reagent (TaKaRa Biotechnology, China) according to the manufacturer’s protocol, cDNA synthesis and qRT-PCR were performed using the Mir-X miRNA First-Strand Synthesis Kit (TaKaRa), SYBR Prime Script RT-PCR Kit (TaKaRa) and QuantStudio 3 realtime PCR system (Life Technologies, MA). Primers are listed in Supplementary Table S1.

**Western blotting**

Western blotting analysis was performed as described previously. Antibodies included β-actin (1:2000, Beyotime), Cleaved Caspase-3(1:1000, Abcam), Cleave PARP (1:1000, Abcam), PPTC7 (1:1000, Abcam), HRP-conjugated goat anti-rabbit IgG (Genshare, China) and HRP-conjugated goat anti-mouse IgG (Genshare, China).

**Luciferase Assay**

Luciferase assay was performed as described previously. The 3’-UTR fragments of human PPTC7 (NM_139283) were amplified by RT-PCR, and inserted into GV272 downstream to the luciferase gene to construct GV272-WT-PPTC7. The putative binding sites for miR-582-5p were mutated by PCR to obtain GV272-mut-PPTC7. For luciferase reporter assay, HEK293T cells were co-transfected with 50 nM of pre-miR-582 mimics or pre-miR-Ctrl, 100 ng GV272-wt-PPTC7 or GV272-mut-PPTC7, and Lipofectamine 2000^TM^ (Invitrogen). After 72 h, cells were harvested and valuated with a Dual-Luciferase Reporter Assay System (Promega).

**Cell viability detect**

Cell viability of NALM-6, KOPN-8 and SUP-B15 cells was determined by using MTT [3-(4, 5-dimethylthiazol-2-yl)-2, 5-diphenyl- 2H-tetrazolium bromide] kit (Beyotime, China) according to the manufacturer’s protocol.

**Supplementary Table S1. PCR Primers Used in the Study.**

| **Name/Target gene** | **Primer sequence** |
| --- | --- |
| Human miR-582-5p | F: 5’- CAGTTGTTCAACCAGTTAC -3’  R: 5’- GAACATGTCTGCGTATCTC -3’ |
| Human miR-582-3p | F: 5’-GCACACATTGAAGAGGACAGAC-3’  R: 5’-TATTGAAGGGGGTTCTGGTG-3’ |
| Human PPTC7 | F: 5’-GACTGCCAGAAGCATTGCTGAG-3’  R: 5’-CGGTGATGTCATCTGGCTTTCC-3’ |
| Human CD276 | F: 5’-CTGGCTTTCGTGTGCTGGAGAA-3’  R: 5’-GCTGTCAGAGTGTTTCAGAGGC-3’ |
| Human β-actin | F: 5’-CACCATTGGCAATGAGCGGTTC-3’  R: 5’-AGGTCTTTGCGGATGTCCACGT-3’ |
| Human-PPTC7 3’UTR | F: 5’-GATCGCCGTGTAATTCTAGAGACTATCATTATTTGTCTTATTAC -3’  R:5’CCGGCCGCCCCGACTCTAGATTAGTATAAATACTATATTTATTAAATATCTTTAC -3’ |

**Supplementary Table S2.** List of human BM samples information

| Human sample number | Gender | Disease type |
| --- | --- | --- |
| 1 | male | unexplained anemia |
| 2 | male | unexplained anemia |
| 3 | male | unexplained anemia |
| 4 | female | unexplained anemia |
| 5 | male | BCP-ALL |
| 6 | male | BCP-ALL |
| 7 | female | BCP-ALL |
| 8 | female | BCP-ALL |
| 9 | female | BCP-ALL |


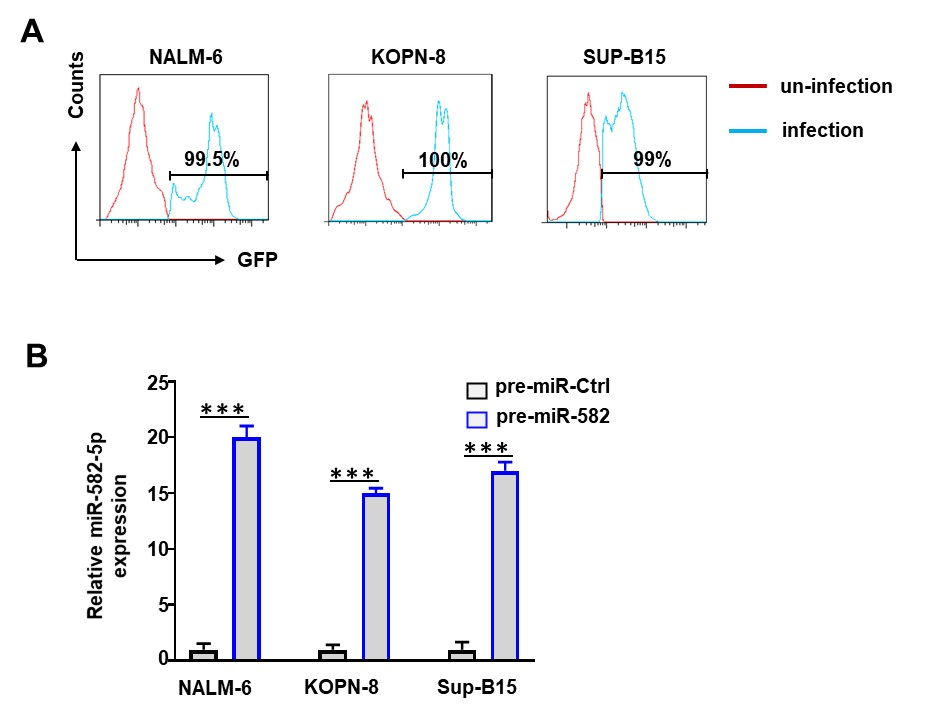


**Fig.S1** Overexpression of miR-582. **(A)** Detect the infection efficiency of EGFP-labeled lentivirus on BCP-ALL cell lines at 24 h by flow cytometry. **(B)** BCP-ALL cell lines were infected with pre-miR-582 or pre-miR-Ctrl lentivirus for 72 h. The expression of miR-582-5p was determined by qRT-PCR. Bars represent means ± SD, ****P* < 0.001.


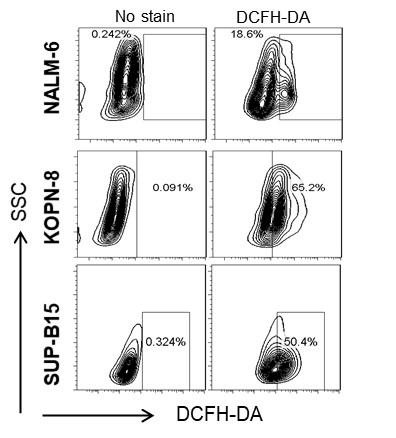


**Fig.S2** Gating strategies. The positive gating of DCFH-DA for single-stained cells with DCFH-DA of figure 3C.


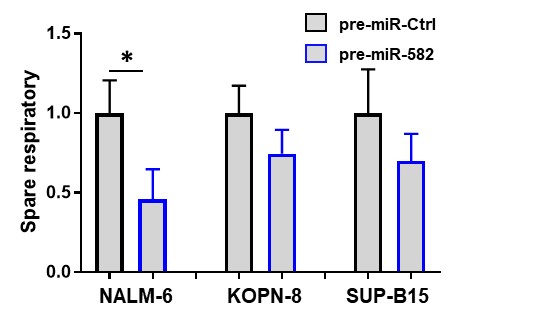


**Fig.S3** The quantification of spare respiratory capacity of the three BCP-ALL cell lines. Bars represent means ± SD, **P* < 0.05.


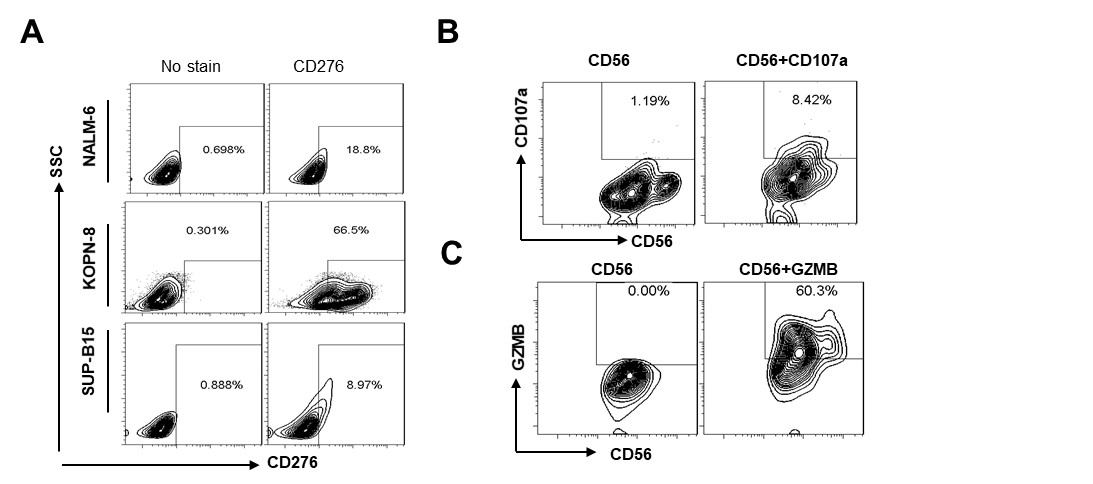


**Fig.S4** Gating strategies. **(A)**The positive gating of CD276 for single-stained cells in BCP-ALL cell lines; **(B)**The positive gating of CD107a for double-stained cells with CD56 and CD107a in NK cells; **(C)** The positive gating of GZMB for double-stained cells with CD56 and GZMB in NK cells.
